# Emerging glyco‐based strategies to steer immune responses

**DOI:** 10.1111/febs.15830

**Published:** 2021-05-15

**Authors:** Marko Anderluh, Francesco Berti, Anna Bzducha‐Wróbel, Fabrizio Chiodo, Cinzia Colombo, Federica Compostella, Katarzyna Durlik, Xhenti Ferhati, Rikard Holmdahl, Dragana Jovanovic, Wieslaw Kaca, Luigi Lay, Milena Marinovic‐Cincovic, Marco Marradi, Musa Ozil, Laura Polito, Josè Juan Reina‐Martin, Celso A. Reis, Robert Sackstein, Alba Silipo, Urban Švajger, Ondřej Vaněk, Fumiichiro Yamamoto, Barbara Richichi, Sandra J. van Vliet

**Affiliations:** ^1^ Chair of Pharmaceutical Chemistry Faculty of Pharmacy University of Ljubljana Slovenia; ^2^ Technical R&D GSK Vaccines S.r.l. Siena Italy; ^3^ Department of Biotechnology and Food Microbiology Warsaw University of Life Sciences‐SGGW Poland; ^4^ Department of Molecular Cell Biology and Immunology Cancer Center Amsterdam Amsterdam Infection and Immunity Institute Amsterdam UMC Vrije Universiteit Amsterdam Netherlands; ^5^ Department of Chemistry and CRC Materiali Polimerici (LaMPo) University of Milan Italy; ^6^ Department of Medical Biotechnology and Translational Medicine University of Milan Italy; ^7^ Department of Microbiology and Parasitology Jan Kochanowski University Kielce Poland; ^8^ Department of Chemistry ‘Ugo Schiff’ University of Florence Florence Italy; ^9^ Division of Medical Inflammation Research Department of Medical Biochemistry and Biophysics Karolinska Institute Stockholm Sweden; ^10^ Vinča Institute of Nuclear Sciences ‐ National Institute of the Republic of Serbia University of Belgrade Serbia; ^11^ Department of Chemistry Faculty of Arts and Sciences Recep Tayyip Erdogan University Rize Turkey; ^12^ National Research Council CNR‐SCITEC Milan Italy; ^13^ Department of Organic Chemistry Faculty of Sciences University of Malaga Spain; ^14^ I3S – Instituto de Investigação e Inovação em Saúde Universidade do Porto Portugal; ^15^ IPATIMUP‐Institute of Molecular Pathology and Immunology Instituto de Ciências Biomédicas Abel Salazar University of Porto Portugal; ^16^ Department of Translational Medicine the Translational Glycobiology Institute Herbert Wertheim College of Medicine Florida International University Miami FL USA; ^17^ Department of Chemical Sciences University of Naples Federico II Complesso Universitario Monte Sant’Angelo Napoli Italy; ^18^ Blood Transfusion Center of Slovenia Ljubljana Slovenia; ^19^ Department of Biochemistry Faculty of Science Charles University Prague Czech Republic; ^20^ Immunohematology & Glycobiology Laboratory Josep Carreras Leukaemia Research Institute Badalona Spain

**Keywords:** autoimmunity, cancer, glycosylation, immune system, vaccination

## Abstract

Glycan structures are common posttranslational modifications of proteins, which serve multiple important structural roles (for instance in protein folding), but also are crucial participants in cell–cell communications and in the regulation of immune responses. Through the interaction with glycan‐binding receptors, glycans are able to affect the activation status of antigen‐presenting cells, leading either to induction of pro‐inflammatory responses or to suppression of immunity and instigation of immune tolerance. This unique feature of glycans has attracted the interest and spurred collaborations of glyco‐chemists and glyco‐immunologists to develop glycan‐based tools as potential therapeutic approaches in the fight against diseases such as cancer and autoimmune conditions. In this review, we highlight emerging advances in this field, and in particular, we discuss on how glycan‐modified conjugates or glycoengineered cells can be employed as targeting devices to direct tumor antigens to lectin receptors on antigen‐presenting cells, like dendritic cells. In addition, we address how glycan‐based nanoparticles can act as delivery platforms to enhance immune responses. Finally, we discuss some of the latest developments in glycan‐based therapies, including chimeric antigen receptor (CAR)‐T cells to achieve targeting of tumor‐associated glycan‐specific epitopes, as well as the use of glycan moieties to suppress ongoing immune responses, especially in the context of autoimmunity.

## Graphical abstract

Glycans are macromolecules that perform crucial roles in development, in cell–cell communication, and in the regulation of immune responses. In this review, we highlight how glycans can be exploited and empowered as treatment or vaccine modalities, particularly in the fight against cancer and autoimmune diseases.

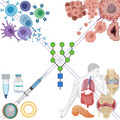

**Abbreviations**
CAR: chimeric antigen receptor
CpG: Cytosine–phosphate–guanosine
DAMP: danger‐associated molecular pattern
DC: dendritic cell
DC‐SIGN: dendritic cell‐specific intercellular adhesion molecule‐3‐grabbing nonintegrin
GlcNAc: *N*‐Acetyl‐D‐glucosamine
GM‐CSF: granulocyte–macrophage colony‐stimulating factor
GMP: Good Manufacturing Practice
HER2: human epidermal growth factor receptor‐2
HIV: human immunodeficiency virus
HNK: human natural killer
I:C: polyinosinic/polycytidylic acid sodium salt
IgG: immunoglobulin G
IgM: immunoglobulin M
IL: interleukin
ITIM: immunoreceptor tyrosine‐based inhibition motif
LPS: lipopolysaccharide
MAG: myelin‐associated glycoprotein
MBL: mannose‐binding lectin
MHC: major histocompatibility complex
MPLA: monophosphorylated lipid A
NK cells: natural killer cells
NLR: NOD‐like receptor
NOD: nucleotide‐binding oligomerization domain
NY‐ESO‐1: New York esophageal squamous cell carcinoma 1
OVA: ovalbumin
PAMP: pathogen‐associated molecular patterns
PD‐1: programmed death 1 receptor
PRR: pattern recognition receptor
RIG‐I: retinoic acid‐inducible gene I
RLR: RIG‐I‐like receptor
SAMPs: self‐associated molecular patterns
Siglec: sialic acid‐binding immunoglobulin‐type lectin
TDB: trehalose‐6,6‐dibehenate
TDM: trehalose‐6,6‐dimycolate
Th: T helper
TLR: Toll‐like receptor
TNF: tumor necrosis factor
Tregs: regulatory T cells

## Introduction

Glycosylation is the most abundant posttranslational modification occurring in mammalian cells and it is markedly changed under pathological conditions, such as cancer and autoimmune diseases [1, 2, 3, 4, 5, 6]. Glycans cover all key immune‐related molecules, such as the major histocompatibility complex (MHC) molecules and the T‐cell receptor, indicating that alterations in cell surface glycosylation can directly affect immune cell function. In addition, immune cells express a wide variety of carbohydrate‐binding receptors, including members of the C‐type lectin receptor, Siglec (sialic acid‐binding immunoglobulin‐type lectin), and galectin families. These receptors decode glycan patterns on host cells, but also on pathogens and are involved in the activation and dampening of immune responses [7]. Strikingly, many immune‐related diseases have been linked to altered glycan profiles [8, 9]. One clear example includes the altered IgG glycosylation observed in the antigen‐binding Fab part of the anticitrullinated protein antibodies (ACPA) in rheumatoid arthritis [10], although the cause or the role of this glycosylation is not yet known. In cancer, major glycosylation‐related changes occur, including the heightened expression of truncated *O*‐glycans, branched *N*‐glycans, diverse fucosylated and sialylated terminal structures, and alterations in glycosaminoglycans and glycosphingolipids [4, 11, 12]. Glycans play a critical role in tumor biology, interfering with cell adhesion molecules, modulating receptor tyrosine kinase activation, and evasion of the antitumor immune response through the interaction with lectin receptors on immune cells [11, 13]. Nevertheless, glycan binding by lectin receptors on immune cells can be exploited for immunotherapy and via glycan‐based therapeutics to improve or correct immunity in cancer, infection, and autoimmunity [14].

Efficient immunity requires not only the proper initiation of responsiveness to harmful infectious agents and malignant cells, but also must instill tolerance to commensal species as well as to innocuous antigens to thereby prevent the development of allergy. If immune reactions proceed unabated after the pathogen has been successfully cleared, patients can develop autoimmunity, resulting in the attack of healthy tissues by the body’s own immune system. In this review, we will discuss the latest developments on the use of glycan‐based tools as a therapeutical approach to activate immune responses in cancer. We will particularly focus on dendritic cells, as these are the most professional antigen‐presenting cells and are master regulators of our immune system. Moreover, we will highlight novel advancements in the field of antiglycan CAR‐T cells and the use of glycans to dampen immune responses in autoimmunity.

## Opportunities and challenges of immunotherapy

In recent years, a number of ‘switches’ that can turn the immune system on or off have been intensively studied and many advances have been made in the field [15, 16, 17, 18]. For example, the discovery of immune checkpoints in the battle against cancer has revealed specific immunosuppressive signaling pathways, such as the interaction between programmed death (PD)‐1 receptor and its ligand PD‐L1 or the inhibitory cytotoxic T‐lymphocyte‐associated protein 4 (CTLA‐4) binding costimulatory molecules on antigen‐presenting cells [19]. The biological importance of these switches is clearly reflected in the successful clinical application of drugs blocking the above‐mentioned pathways, which overcome immunosuppression by certain tumors, resulting in improved overall survival of patients [20, 21]. In contrast, the use of classical immunosuppressants, such as glucocorticoids and others, is very frequently associated with excessive general suppression and risk of life‐threatening infections [22].

The design of novel immunomodulatory approaches must therefore be aimed at increased specificity and efficacy [23], by targeting specific cells or molecular pathways to reduce side effects of treatment by regulating only selected processes. However, before clinical application, several factors for predicting the final immunological outcomes must be considered, including potential target cells and their function, biochemical pathways involved, amplification of induced changes by positive feedback loops, negative feedback or regulatory mechanisms, and summation of exerted effects.

### Plasticity of dendritic cells in controlling innate and adaptive immune responses

**Fig. 1:**
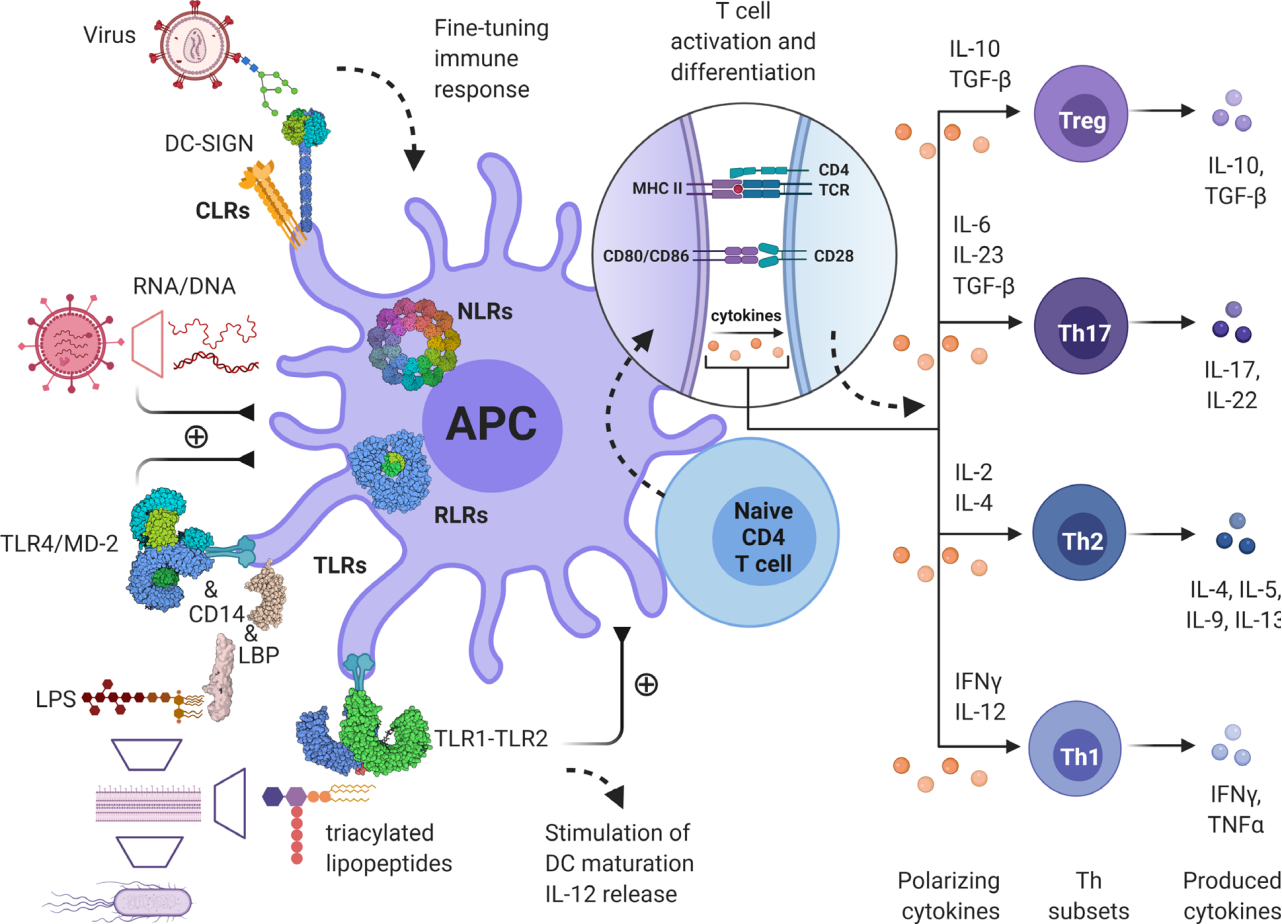
Innate immunity and glycans play a key role in immune responses and homeostasis. Antigen‐presenting cells (APCs), such as dendritic cells and macrophages, recognize pathogens using PAMPs and DAMPs via a group of so‐called pattern recognition receptors, the most important families of these receptors being TLRs, nucleotide‐binding oligomerization domain NLRs, RIG‐I‐like receptors (RLRs), and C‐type lectin receptors (CLRs). Glycans are often key part of these interactions as well as of the following interplay between innate and adaptive immunity, thus fine‐tuning immune responses, for example, by differential stimulation of T helper (Th) cell activation and differentiation to individual cellular subsets, including regulatory T cells (Tregs) with varying functions.

Dendritic cells (DCs) are the most superior antigen‐presenting cells of our immune system and as such the master regulators of both immunity and tolerance (Fig. 1) [24]. Seeded in virtually all tissues as immature DCs, these cells patrol their surroundings for incoming pathogens or signs of tissue damage. To achieve this task, DCs are equipped with an array of pattern recognition receptors (PRRs), including Toll‐like receptors (TLRs), nucleotide‐binding oligomerization domain (NOD)‐like receptors (NLRs), retinoic acid‐inducible gene I (RIG‐I)‐like receptors (RLRs), and C‐type lectin receptors. These receptors are specific for conserved microbial structures, called pathogen‐associated molecular patterns (PAMPs), but can also recognize danger‐associated molecular patterns (DAMPs), molecules released from necrotic tissue [25]. Based on their ability to dampen immune responses, glycans have been proposed to act as self‐associated molecular patterns or SAMPs [26]. Nevertheless, also pathogen‐exposed glycans can bind the glycan‐binding C‐type lectins and thus participate in the inflammatory response toward the pathogens.

After triggering of PRRs by PAMPs or DAMPs, DCs undergo drastic biological changes, termed maturation, which result in extensive expression of costimulatory molecules, production of various pro‐inflammatory biomolecules (cytokines), and migration to secondary lymph nodes. Importantly, depending on the type of maturation signals, DCs can greatly adjust the amount and type of cytokines or costimulatory molecules they express and can also express inhibitory molecules and anti‐inflammatory cytokines, leading to tolerance induction (Fig. 1) [27]. The abundance of various receptors that regulate efficient DC responsiveness is reflected in the fine‐tuning of DC activation, which depends on the type of PAMPs and consequent binding of various PRRs or their combinations, resulting in different ‘flavors’ of the DC activation state capable of inducing different classes of immune responses [27]. One well‐known example is the simultaneous activation of TLRs and C‐type lectin DC‐SIGN by mannose‐containing PAMPs [28, 29], which, for example, are carried by HIV‐1 [30]. By concomitant DC‐SIGN activation, HIV can dampen TLR‐induced DC maturation, resulting in increased production of the immunosuppressive interleukin (IL)‐10 [31]. This outstanding functional plasticity extends well beyond activation of various effector subsets such as Th1‐, Th2‐, or Th17‐type effector T cells [32], but can also lead to immune tolerance by induction of regulatory T cells (Tregs) [33]. It is worth noting that while pathogens directly stimulate DCs, they can also stimulate other immune cells (NK cells, Tregs, etc.) and tissue cells, which can in turn also influence DC activation and the immunological outcome.

With possibly few exceptions, almost all immune cell subsets are subjected to functional plasticity, which is greatly dependent on their microenvironment, cell‐to‐cell interactions as well as several other factors. A textbook example is the activation and polarization of naive T cells upon antigen encounter, which can give rise to the development of either Th1, Th2, Th17, and other effector subtypes or even to induction of various regulatory T‐cell subsets (Fig. 1) [34, 35, 36]. Perhaps the greatest functional plasticity belongs to DCs that play a major role in shaping the adaptive immune responses and are central to optimal and successful vaccination outcomes. The immunogenic vs. tolerogenic effect of DCs is largely dependent on their activation state. Indeed, strong immunogens, such as TLR ligands (agonists), as well as certain pro‐inflammatory cytokines, can be considered strong primers of DC maturation, their migration to secondary lymph nodes, and thereafter their induction of various T‐cell effectors. However, there are known examples where DCs do not reach their full maturation, leading to a so‐called ‘semi‐mature’ state, favoring the induction of tolerance over immunity [37]. Semi‐maturation entails similar expression profiles of costimulatory molecules; however, such cells lack other signals necessary for the induction of immune responses, namely appropriate cytokine production crucial for T‐cell polarization. Inadequate maturation can occur for example by certain microbes and their components, for example, lactobacilli from the gut flora [38], or by maturation induced by tumor necrosis factor (TNF)‐α alone [39] or importantly, TLR ligands at suboptimal concentrations [40]. In such instances, semi‐matured DCs favor the induction of Th2 or regulatory T‐cell responses. DC maturation can also be thwarted by immunosuppressive components present in their microenvironment. Most likely, increased presence of certain endogenous or exogenous immunosuppressants, such as corticosteroids (e.g., dexamethasone) or the active metabolite of vitamin D3 (vit D_3_), can greatly inhibit the DC maturation process and cause the so‐called ‘alternative activation’, wherein the DCs display extensive expression of inhibitory, instead of costimulatory, surface molecules and have increased production of immunosuppressive cytokines, such as IL‐10 [41, 42]. The presence of such components can also be associated with a specific anatomical region like for example the gut mucosa, where the increased presence of certain dietary components like vitamin A can favor the induction of tolerogenic DC subsets capable of inducing regulatory T cells [43].

The tight balance between immunity and tolerance is crucial for maintaining immune homeostasis. An exarcerbated immune reaction, without proper immune resolving, can lead to unwanted immunity to self‐tissues (autoimmunity) or innocuous antigens (allergy). In contrast, an inadequate immune response may allow immune escape of transformed cells and the development of cancer. In summary, both induction of immunity and tolerance are extensively regulated and fine‐tuned by a great number of factors, which need to be considered when attempting to manipulate the immune system. Besides choosing the specific molecular target present on immune cells, one should pay additional attention to the tissue environment, meeting the criteria for optimal immune cell maturation and take into account potential immune cell subtypes, which could express the molecular target in question but would respond according to their pre‐determined biology.

### Targeting of lectin receptors on antigen‐presenting cells in vaccination approaches

Vaccine design has been greatly inspired by the process of antigen delivery/presentation [44]. For successful vaccination purposes, efficient antigen capture and presentation are a prerequisite for adequate helper and cytotoxic T‐cell responses. Most C‐type lectins recognize and bind glycosylated antigens, which generally results in internalization and processing for loading onto MHC molecules and subsequent presentation to antigen‐specific T cells. Using this rationale, C‐type lectin receptors can be targeted both *in vitro* and *in vivo* for increased antigen delivery [45]. One of the most studied C‐type lectins is perhaps DEC‐205, a C‐type lectin expressed on mouse and human DCs. In particular, DEC‐205 was successfully targeted using tumor antigens conjugated to anti‐DEC‐205 monoclonal antibody, thereby significantly enhancing antitumor immunity [46, 47, 48]. In addition, anti‐DEC‐205 conjugates have also been tested in immunization protocols against viruses [49]. Similar observations of increased T‐cell responses emerged in studies where the C‐type lectins, Clec9A and Clec12A, were targeted [50, 51, 52, 53]. Other important C‐type lectins involved in antigen presentation are the mannose receptor, dendritic cell‐specific intercellular adhesion molecule‐3‐grabbing nonintegrin (DC‐SIGN), Langerin, DCIR, and Dectin‐1. While most attempts of targeting antigens to C‐type lectins were performed using conjugates with monoclonal antibodies, glycan‐based ligands are a viable alternative. This approach has been widely exploited in some studies involving Dectin‐1, mannose receptor, DC‐SIGN, and others [54, 55, 56, 57, 58, 59, 60]. Since C‐type lectin members contain various signaling domains and are represented on different types of antigen‐presenting cells, the design of novel specific glycan‐based ligands could be useful in targeting specific cell types, which in turn can define the class of T‐cell responses [45]. Indeed, the targeting of specific antigen‐presenting cell types has already been proven to effectively induce regulatory T‐cell responses [61]. In this way, C‐type lectin receptor‐specific antigen delivery could also be useful in inducing tolerance instead of immunity.

In this review, we will discuss recent advances on the use of glycans as a targeting device to manipulate immune responses toward immunity or tolerance in the treatment of cancer and autoimmunity. We will address how glycans can be employed as a targeting moiety, but also how glycan‐derived and glycan‐modified nanoparticles can aid in the steering of immune responses. Finally, we will highlight some of the latest developments regarding the use of glycan‐directed chimeric antigen receptor (CAR)‐T cells and discuss some potential applications of glycan nanodevices.

## New glyco‐based strategies to steer immune responses in infection, cancer, and autoimmunity

### Improving immune responses using glycan‐modified nanoparticles and cells

**Fig. 2:**
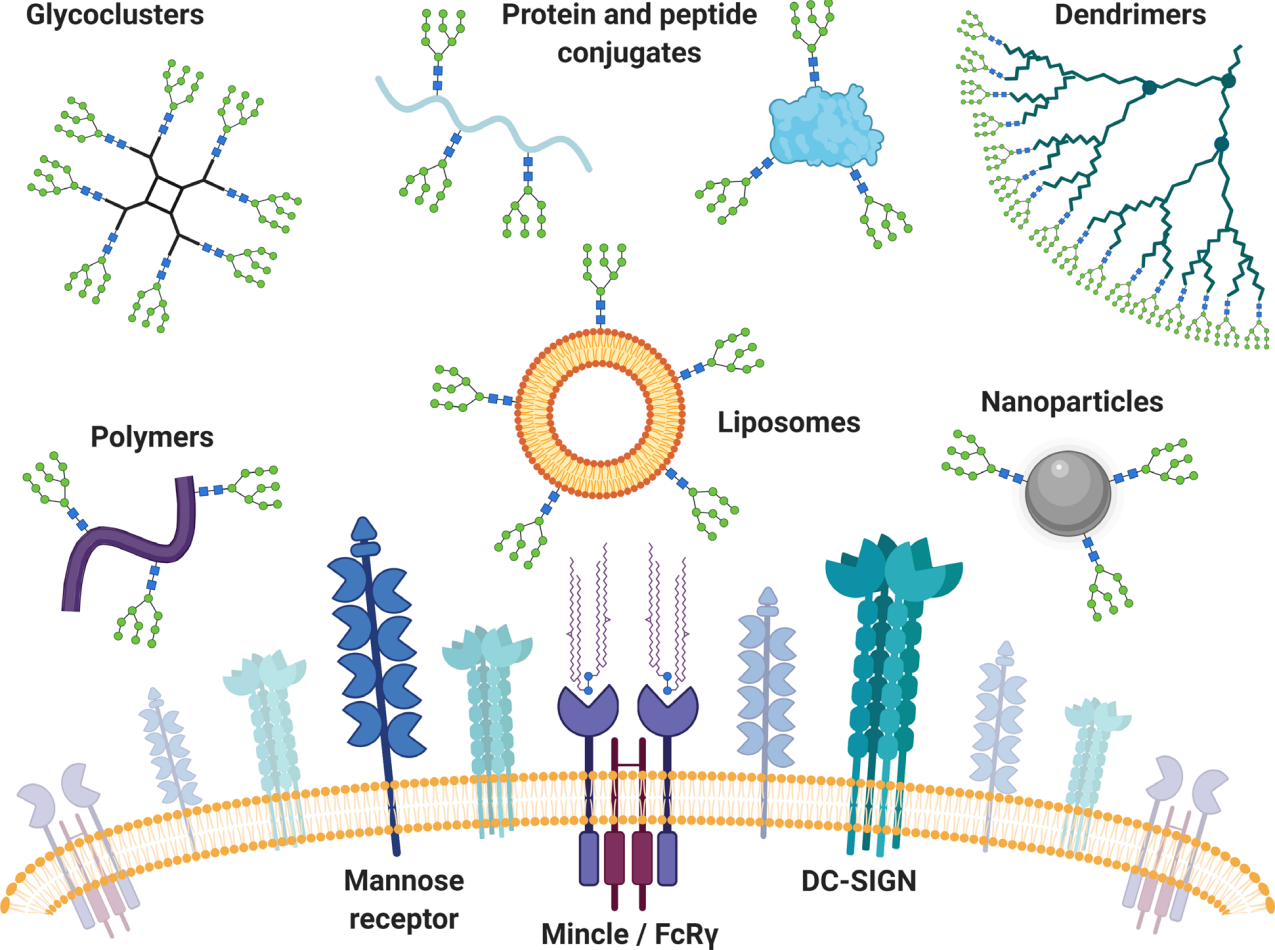
Overview of carrier systems used for multivalent glycan display. Multivalent high‐mannose glycan presentation on glycoclusters, polymers, antigens (proteins and peptides), liposomes, dendrimers, and nanoparticles has been successfully used for targeting C‐type lectin receptors, like DC‐SIGN, Mincle, and mannose receptor. In contrast, the C‐type lectin Mincle associates with the signaling adaptor FcRγ, which upon binding of its ligand microbial cord factor trehalose‐6,6‐dimycolate directly triggers pro‐inflammatory responses. Therefore, the adjuvanticity of Mincle ligands is widely studied within the field of vaccinology. Adapted from [67].

An uncontrolled growth and a resistance to apoptosis are some of the characteristics hallmarks of cancer. At early stages, the immune system is able to control tumor growth; however, as the tumor progresses, some tumor cells escape immune surveillance and are able to expand and metastasize to distinct sites to establish novel tumor nodules. The recent success of immune checkpoint blockade, using anti‐PD‐1, PD‐L1, or CTLA‐4 antibodies, has demonstrated the power of the immune system in fighting cancer [62]. Yet, some patients relapse or do not even respond to this treatment, suggesting the existence of additional immune evasion strategies or the absence of effective tumor‐specific adaptive immunity. The targeting of DCs is an efficient way to improve T‐cell activation in cancer immunotherapy [63]. DC vaccines or antigen‐pulsed DCs can induce antigen‐specific T‐cell response *in vivo* [64], but there is still much to understand for the development of this type of vaccine. Nanoparticle‐based approaches have been proposed to enhance DC targeting, deliver immunomodulators to program DCs, improve antigen stability, and allow for co‐delivery of adjuvants and other molecules of interest on the same nanoplatform (Fig. 2) [65, 66].

#### C‐type lectin targeting for tumor antigen delivery

The recognition and internalization of carbohydrate‐containing antigens by DCs are usually mediated by C‐type lectin receptors [68], such as mannose receptor and DC‐SIGN [45]. Nevertheless, many other C‐type lectins, like the Mincle receptor, may be targeted through modified natural ligands (Fig. 2) [69]. Although C‐type lectins can be targeted through lectin‐specific antibodies, we will discuss in this section solely the use of natural (glycan) ligands as the targeting moiety, focussing the model C‐type lectins, DC‐SIGN, mannose receptor, and Mincle (Fig. 2). Glycan recognition by C‐type lectins has been recently reviewed [70].

The mannose receptor has been a prototype lectin in vaccine studies for decades as a target receptor for many different mannosylated compounds. These mannosylated compounds have been used in different disease settings, including cancer, allergy, and infection [71, 72]. Coupling of monovalent or multivalent mannosides to lipopeptides [73] or proteins also directs [74, 75, 76] antigens to DCs and enhances uptake by the mannose receptor. In a study on mannosylated MUC1 tandem repeat peptides, the divalent mannosylated peptide was superior in binding and induction of MUC1‐specific antibodies compared to the monovalent mannosylated MUC1 peptide [74]. Mannosylation with synthetic long constructs specifically promoted antigen cross‐presentation and CD8^+^ T‐cell activation [77], showing that glycans can indeed skew immune responses into the desired direction.

Mannose‐functionalized nanoparticles have been employed as the targeting vehicle to reach DCs *in vivo* [78]. Chitosan nanoparticles loaded with whole‐cell tumor lysates and modified with mannosylated groups were able to enhance uptake by murine DCs, resulting in a delay in melanoma tumor growth due to augmented CD8^+^ T‐cell responses [79]. Also, mannan‐coated PGLA nanoparticles were able to enhance CD4^+^ and CD8^+^ T‐cell responses, in this case to the model antigen ovalbumin (OVA) [80].

Recently, a DNA vaccine‐containing liposomes loaded with MART‐1 tumor antigen DNA and a di‐shikimoyl mannose mimic were generated [81]. Upon injection, these liposomes were efficiently taken up by CD11c^+^ DCs and induced long‐lasting antitumor immunity in a prophylactic murine melanoma model. Also, in a therapeutic setting this vaccine could delay tumor growth and prolong survival of the mice. Another study proposed the use of glycan‐modified liposomes to target tumor‐associated macrophages [82], which play a fundamental role in promoting tumor growth and metastasis when they acquire a polarized M2 phenotype [83]. Since tumor‐associated macrophages overexpress on their surface mannose receptors [84], mannose‐functionalized liposomes were synthesized to study the effect of these glycan‐nanostructures on tumor‐associated macrophage activation and polarization. Indeed, high internalization of mannose‐functionalized liposomes was observed in M2 macrophages compared to M1. Interestingly, mannose‐liposomes were able to induce polarization of M0 and M2 to an M1 phenotype, which was associated with the expression of a specific costimulatory molecule, CD86 that boosts an immunological response against the malignancy. The surprising ability of mannose‐liposomes to induce the shift from M2 to a M1 phenotype underlines that glycan‐liposomes could actually selectively eliminate or re‐educate tumor‐associated macrophages, which represents a fascinating goal in cancer immunotherapies.

The above studies clearly demonstrate the power of targeting mannose receptor (for an complete overview, we refer the reader to a more comprehensive review [85]), yet few mannosylated compounds have made it to the clinic so far. An exception is the FDA‐approved ^99^Tc‐tilmanocept (LYMPHOSEEK®, consisting of a diethylenetriaminepentaacetic acid (DTPA)‐mannosyl‐dextran backbone), which efficiently targets the mannose receptor on macrophages and DCs in lymph nodes and is thus used as a detection agent in sentinel lymph node procedures [86] in oral and breast cancer, as well as melanoma [87, 88]. From an immunotherapeutic setting, a 15‐year follow‐up on clinical trials employing oxidized mannan–MUC1 revealed that treatment with the oxidized mannan–MUC1 significantly reduced the recurrence rate in stage II breast cancer patients [89].

Natural ligands for C‐type lectin DC‐SIGN constitute high‐mannose oligosaccharides and Lewis‐type moieties, such as Lewis x (Le^x^) and Lewis b (Le^b^). Since the recognition of carbohydrate antigens is mediated by the carbohydrate recognition domain of DC‐SIGN [90, 91], globular nanoparticles mimicking the physiological glycoproteins of pathogens have been envisaged as a smart platform to target DC‐SIGN. In particular, Le^x^ or Le^b^ functionalized liposomes have been exploited to target murine and human DCs. Phosphatidylcholine/phosphatidylglycerol liposomes, conjugated to Le^x^ and Le^b^ via maleimide‐thiol chemistry, were able, in the presence of LPS, to induce CD4^+^ and CD8^+^ T‐cell activation 100‐fold better than nonmodified liposomes [92]. Such results are highly promising in the development of new smart glycan‐based nanoplatforms, showing how crucial the role of glycan‐based liposomes in immunotherapies for the treatment of tumors can be. Nevertheless, the highest affinity C‐type lectin ligands may not be the best targeting devices. Using a systemic library of multivalent mannosides conjugated to the melanoma‐associated gp100 antigen and a TLR‐7 agonist, Li *et al*. investigated the binding characteristics to DC‐SIGN and their ability to induce antigen presentation [93]. Strikingly, the compound with the highest affinity for DC‐SIGN, the α‐1,2‐di‐mannoside cluster, actually hampered T‐cell activation.

Also, the antigen formulation, multivalency, and delivery vehicle are crucial for the type of response induced. Where DC‐SIGN prefers virus size particles/liposomes, Langerin actually is best targeted with smaller glycated peptide antigens [58]. Recently, branched polyamidoamine (PAMAM) dendrimers carrying multiple copies of the melanoma gp100 synthetic long peptide and the shared DC‐SIGN and Langerin ligand Lewis y (Le^y^) have been prepared as intradermal antitumor vaccine carrier [94]. These glycan‐functionalized dendrimers were internalized by skin‐resident DCs and were able to stimulate gp100‐specific CD8^+^ T‐cell responses. Moreover, inclusion of adjuvant may alter the DC maturation status, the way antigens are handled after uptake by the C‐type lectin and thus the outcome of the induced immune response. For instance, concomitant TLR4 triggering promotes cross‐presentation to CD8^+^ T cells after antigen uptake by DC‐SIGN [95]. Also when targeting the mannose receptor, accommodating TLR ligands enhances the uptake of the vaccine and subsequently promote the differentiation of Th1 cells [96]. As further proof of the value of DC‐SIGN targeting for antigen delivery, more recently, virus‐like particles (VLP) were conjugated to either aryl mannoside residues and an OVA peptide antigen [97]. The construct proved to induce antigen‐specific immune responses (i.e., activation of CD4^+^ cells and cytokine release) in mice.

The C‐type lectin Mincle is widely expressed on myeloid cells, including monocytes, macrophages, and DCs [98]. Mincle acts both as a pathogen receptor, by recognizing among others fungal [99] and mycobacterial [100] ligands, and as a sensor of death cells through the interaction with SAP130 released from dying cells [101]. Mincle associates with the FcRγ‐chain and therefore differs from mannose receptor and DC‐SIGN in its ability to directly activate cells without the need of concomitant PPR signaling. Mincle signaling triggers the SYK‐CARD9‐Bcl10 axis, leading to NF‐κB activation and production of pro‐inflammatory cytokines [101] and shifts macrophage differentiation toward a M1‐like phenotype [102]. This unique characteristic of Mincle provides the rationale for the adjuvanticity of the microbial cord factor trehalose‐6,6‐dimycolate (TDM, Fig. 2) and its synthetic analogue trehalose‐6,6‐dibehenate (TDB) [103] and empowers Mincle as a prime target for vaccination purposes.

In their key study, Decout *et al*. investigated the role of the fatty acid tails in TDM, showing that they are crucial for the binding to the Mincle carbohydrate recognition domain [104]. Based on the molecular dynamics simulations, novel glucose derivatives acetylated with 2‐tetradecyloctadecanoic acid (GlcC14C18) and mannose derivatives (mannose 2‐tetradecyloctadecanoate, ManC14C18) were synthesized. Both compounds induced high levels of TNFα in mouse and human DCs and macrophages and strong Th1 and Th17 responses *in vivo*. Indeed, several studies have identified novel TDM and TDB analogues that have superior agonistic activity [105, 106].

Trehalose diamides and sulphonamides, as well as trehalose diester with shortened acyl chains, appear more potent than the TDB and TDM compounds in evoking Mincle signaling, cytokine secretion, and steering of immune responses [107, 108]. Synthetic TDM derivatives with improved physiochemical properties have been used to functionalize silica nanoparticles with a superparamagnetic iron oxide core (to improve separation of the product) [109]. These nanoparticles were able to trigger Mincle activation in reporter cell lines and also evoked TNFα and IL‐6 secretion in mouse RAW264.7 and human PBMC, respectively. Combinations of Mincle ligands with other PPR stimuli, such as Poly I:C [110] or bis‐(3'‐5')‐cyclic dimeric guanosine monophosphate (c‐di‐GMP, a ligand for the STING receptor) [111], may even provide superior memory responses and long‐lasting cellular and humoral responses, as demonstrated in cattle and pigs. Incorporation of both TDB and monophosphoryl lipid A (MPLA) enhanced the efficacy of a *Mycobacterium tuberculosis* subunit vaccine, providing long‐term protection against a mycobacterial challenge [112].

To date, novel Mincle agonists are still being identified and tested for their immune‐activating properties, although structural requirements might differ for mouse and human Mincle [113], warranting caution in evaluating the adjuvanticity for Mincle *in vivo* in mice. These include among others, the S‐layer glycoprotein from *Lactobacillus kefiri* [114], whose adjuvant activity was dependent on the glycan components and on the presence of Mincle and CARD9 in mouse DCs. Brartemicin, a glycosyl glycoside derivative isolated from *Nonomuraea* species with similarity in structure to TDB, was originally identified for its antitumor activities and is also recognized by Mincle [109, 115]. Lipidated Brartemicin analogues and especially the *o*‐substituted variant showed strong inflammatory activities, inducing Mincle signaling and pro‐inflammatory cytokine responses in both mouse bone marrow‐derived macrophages and human monocytes [116, 117].

Together, these results indicate that a thorough design of C‐type lectin receptor‐targeting glycans and their configuration is absolutely required to obtain the most optimal vaccine formulation. The development of C‐type lectin‐specific glyco‐mimetics will expand the tools glyco‐chemists can employ in their design to create more versatile and C‐type lectin‐specific targeting moieties and platforms [118].

#### Glycoengineering approaches for vaccination purposes

The targeting of C‐type lectins is not the only, exclusive way in which glycan‐modified products can be employed for vaccination purposes. Glycosylation not only directs the folding of a certain glycoprotein, it can also control its biological activity, potency, and pharmacokinetic properties; therefore, the glycoengineering of biologicals is gaining more attention. Manipulation of the cellular glycosylation machinery will thus allow for production of recombinant proteins with preferred glycosylation patterns.

The early, pioneering work using glycosylation mutants of Chinese hamster ovary (CHO) cells has been instrumental to decipher the role of individual glyco‐genes and has yielded enormous insight in the biological processes mediated by glycans [119]. These glycoengineered cells have been widely used already for decades in the production of therapeutical proteins with a defined glycosylation pattern [120]. In the last decade, technical advances in the field of genetic engineering have revolutionized the possibilities to modify the glycosylation machinery of cells. Especially, the use of CRISPR/Cas to abolish or induce expression of glycosyltransferases or other glycosylation‐related genes has opened up novel possibilities to manipulate the cellular glycome [121, 122]. To date, a whole validated set of CRISPR guideRNAs (GlycoCRISPR) has been published to facilitate to glyco‐editing of human cells [123]. Below, we will highlight how glyco‐editing can aid in the optimization of biological agents.

Lysosomal storage diseases are metabolic disorders, arising from group of single gene defects and characterized by lysosomal dysfunction and an inability to degrade certain proteins, lipids, or oligosaccharides. Enzyme‐replacement therapy is often the treatment of choice, yet the targeted delivery of the enzymes is crucial for obtaining the desired therapeutic efficacy. Early work by Furbish *et al*. revealed that treating the rat glucocerebrosidase with neuraminidase enhanced uptake by the Ashwell–Morell C‐type lectin in hepatocytes, while β‐galactosidase and β‐*N*‐acetylglucosaminidase treatment promoted the uptake by Kupfer cells [124]. In patients with Gaucher's disease that lack a functional glucocerebrosidase, the injection of macrophage‐targeted glucocerebrosidase was later shown to induce objective clinical responses, reversing the progression of the disease [125]. Recently, a CRIPSR‐based screen was conducted in CHO cells to manipulate *N*‐glycosylation and mannose‐6‐phosphate processing of α‐galactosidase A, the enzyme defective in Fabry disease [126]. Strikingly, depending on the glycoforms tested, α‐galactosidase A showed differential targeting to the heart, spleen, liver, or kidney. Moreover, α‐galactosidase A decorated with α2‐3‐linked sialic acids had an enhanced half‐life and displayed an improved biodistribution compared to the α‐galactosidase A with α2‐6‐linked sialic acids. Such unbiased screens could clearly open up new possibilities to advance existing enzyme replacement therapies for lysosomal storage diseases.

In immunity, glycosylation has a predominant role in regulating Fc receptor binding and antibody effector responses and altered IgG glycosylation patterns are frequently observed during inflammation, infection, or autoimmune diseases [127, 128]. The Fc‐domain of IgG contains a conserved *N*‐glycosylation site that is most commonly covered with a biantennary *N*‐glycan that can carry a bisecting GlcNAc, be core‐fucosylated and/or galactosylated and sialylated [127]. Changes in Fc‐glycosylation and altered biological function were first reported by Jeffrey Ravetch, who, in 2006, demonstrated that the anti‐inflammatory properties of intravenous IgG or Fc fragments were due to differential sialylation of the Fc‐glycan [129]. Strikingly, these anti‐inflammatory effects are only observed on Fc‐glycans carrying α2‐6‐linked sialic acids, while IgGs with α2‐3‐linked sialic acids do not share these characteristics [130]. In addition, administration of solubilized glycosyltransferases B4GALT1 or ST6GAL1, that respectively add galactose or sialic acid, was able to convert pathogenic IgGs into anti‐inflammatory IgGs, thereby attenuating autoimmune inflammation [131]. Besides sialylation, also other IgG glycoforms impact the biological activity of the IgG. For instance, a lack of core fucosylation enhances binding to the FcγRIIIa and promotes antibody‐dependent cellular cytotoxicity, while galactosylated IgG glycoforms bind C1q and thereby initiate complement activation (nicely reviewed in Ref. [132]). Clearly, translation of this knowledge to the clinical‐grade monoclonal antibodies could enhance their efficacy, while reducing safety concerns or manufacturing costs. Obinutuzumab (anti‐CD20) is probably the first of many glycoengineered antibodies that have found its way to the clinic and is currently used for the treatment of B‐cell lymphomas [133].

Next to therapeutic antibodies, also other glycoproteins may be glycoengineered to enhance their biological activity. Interesting in this respect are cytokines, whose glycosylation status regulates receptor binding, biological stability, and function [134]. Glycoengineered versions of interferon‐α and ‐β have been developed that show enhanced pharmacokinetic properties and prolonged signaling capacities [135, 136]. Glycosylated forms of interferon‐β are currently used in the clinic for the treatment multiple sclerosis [137]. Also different glycoforms of IL‐6 [138] and granulocyte colony‐stimulating factor [139] have been synthesized for research and treatment purposes.

Finally, whole cells may undergo genetic remodeling to express the desired glycosylation pattern. Especially in oncology, whole tumor cell vaccines are an attractive immunotherapy strategy to induce strong cytotoxic T‐cell responses toward the tumor. In this context, the GVAX vaccine, consisting of inactivated tumor cells transduced with the *GM‐CSF* gene, is still undergoing phase I and II clinical trials in several cancer types [140]. The pancreatic GVAX vaccine could induce antibodies to tumor‐associated glycans [141], suggesting that manipulation of the tumor cell glycome could potentially augment this response. Indeed, kifunensine treatment of melanoma cells enhanced the uptake of apoptotic tumor vesicles by DCs and boosted melanoma‐specific T‐cell responses, showing that not only humoral, but also cellular responses could benefit from glycoengineering of the tumor cell vaccines [142]. Glycoengineering is of course not limited to cancer immunotherapy, but extends to infectious diseases and even the dampening of unwanted inflammatory or autoimmune conditions (discussed below).

Overall, modifying the glycosylation of therapeutic recombinant monoclonal antibodies, glycoproteins, or even whole‐cell vaccines is an attractive possibility to improve newly developed or existing biologicals, thereby optimizing their use in clinical practice or in vaccination strategies.

#### Glycan‐based nanoparticles as delivery platforms

In addition to delivering tumor antigens to antigen‐presenting cells, nano‐approaches could help in potentiating antitumor responses in combination with PD‐1 blockade and OX40 co‐stimulation. In this respect, polysaccharide polymers as delivery systems in *in vivo* targeting of DCs have attracted growing interest [143]. Polysaccharide‐based nanoparticles, usually prepared from natural polymers, such as alginate, chitosan, and its derivatives, cyclodextrin, hyaluronic acid, inulin, pullulan, and their combinations, have gained attention, because they are often: (a) abundantly available and relatively inexpensive; (b) safe, nontoxic, and nonreactogenic; and (c) have a good stability, hydrophilicity, biocompatibility, and biodegradability. In addition, polysaccharides can be easily functionalized by (bio)chemical means due to the presence of several reactive groups in their structure and some of them can provide targeting mechanisms due to receptor recognition and binding, mucosal adhesion and transport, site‐specific enzymatic degradation, and environmental triggering. Some of these materials are inherently immunogenic and can also act as an adjuvant.

In this regard, chitosan, a linear amino polysaccharide obtained by a partial deacetylation of chitin, is one of the most promising polysaccharide polymers. It is a nontoxic, biodegradable, biocompatible, and muco‐adhesive polymer which was approved as Generally Recognized as Safe by the US Food and Drug Administration. However, chitosan is poorly soluble and precipitates at physiological pH; thus, several derivatives have been widely investigated, such as phosphorylated and mannosylated chitosan and N‐trimethyl chitosan (TMC). Some examples of experiments on targeting DCs *in vivo* with antigen‐loaded nanoparticles using chitosan and its derivatives are as follows: immunization against viral influenza A, hepatitis B and bacterial *S. equi*, and model antigens (OVA, urease) in immunological studies, as well as toxoids of tetanus and diphtheria [144].

Table 1 summarizes *in vivo* experiments with antitumor vaccine candidates using polysaccharide‐based particulate delivery systems for targeting DCs. For all tabulated DC vaccinations, nanoparticles engineered from various polysaccharide polymers were used in nanoparticle‐mediated delivery to DCs of antigenic substances produced in tumor cells. In some cases, to further enhance immunogenicity, antigens were co‐delivered with an adjuvant.

**Table 1:** Polysaccharides used as nanoparticle carriers.

Polysaccharides for nanoparticles	Tumor antigen/adjuvant	Study type	Outcome	Ref.
Cholesteryl pullulan	HER2 protein	HER2‐expressing cancer patients, clinical trial	Vaccine was well tolerated and induced antigen‐specific immune responses	[145, 146]
Cholesteryl pullulan	NY‐ESO‐1 protein	NY‐ESO‐1‐expressing tumor patients, clinical trial	Vaccine induced antigen‐specific immune responses	[147]
Hyaluronic acid	Poly‐l‐lysine /CpG	E.G7‐OVA lymphoma, mouse model	Increased growth inhibition and a strong antitumor memory response	[148]
Hyaluronic acid	OVA peptide 257‐254	T1 cervical cancer, mouse model	Substantial inhibition of tumor growth	[149]
Chitosan	Whole cancer cell lysates, mannose	B16 melanoma, mouse model	Increased tumor growth inhibition	[79]
Alginate	OVA peptide 323‐329	B16 melanoma, mouse model	OVA peptide inhibited tumor progression more effectively than using the peptide alone.	[150]
γ‐PGA/chitosan	MUC1 protein	MUC1‐expressing cancers, mouse model	High‐level immune response and improved immunogenicity	[151]
Mannosylated alginate	OVA	E.G7‐OVA lymphoma, mouse model	Major cytotoxic response and increased tumor growth inhibition	[152]
Chitosan	OVA/poly I:C	EG.7 and TC‐1, mouse model	Greater antitumor efficacy in EG.7 and TC‐1 tumor‐bearing mice compared to the control	[153]

Attempts have also been made with nanoparticles, which use carbohydrates as targeting moiety to improve the antigen presentation by antigen‐presenting cells. Indeed, anticancer peptide‐based vaccines offer some advantages as the synthesis and purification can nowadays be carried out in automated fashion, and the scale‐up in Good Manufacturing Practice (GMP) conditions is usually possible. On the other hand, the peptide selection process is based on challenging MHC fitting (furthermore, tumor‐driven downregulation of MHC class I molecules often occurs) and peptides are susceptible to degradation *in vivo*. A strategy to improve the performance of anticancer peptide vaccines is to use suitable delivery systems [154]. Among the myriads of nanosystems that have been proposed, the glycosylated ones have often been tested in order to improve the uptake by antigen‐presenting cells. This is the case of gold nanoparticles coated with simple β‐d‐glucosides and a highly immunogenic peptide that contains a cytotoxic restricted epitope of the bacterial pathogen *Listeria monocytogenes*, namely listeriolysin O 91–99 peptide (LLO_91–99_), that were challenged in vaccination of mice bearing melanoma [155]. This construct was designed based on the fact that the LLO_91–99_ peptide was able to reduce metastasis in mice when formulated in a DC‐based vaccine. The nanotherapy based on GNP‐LLO_91–99_ induced tumor apoptosis and melanoma‐specific cytotoxic Th1 responses, with a similar performance as DC‐GNP‐LLO_91–99_ in terms of reducing tumor size, but with a better performance than DC‐LLO_91–99_ vaccines. Furthermore, the adjuvant activity for recruiting and activating DCs was demonstrated, confirming the possibility to avoid *ex vivo* loading of DCs.

Lipid‐calcium‐phosphate was used for encapsulation of a derivative of tyrosinase‐related protein 2 peptide (Trp2_180–188_), a relatively poor immunogenic peptide, and used in cancer immunotherapy [156]. Calcium phosphate (CaP) cores worked as aluminum salt‐like adjuvant and were protected with dioleoylphosphatidic acid. Cationic dioleoyl‐3‐trimethylammonium propane/cholesterol salts were employed to generate stable nanoparticles together with the insertion in the outer shell of 1,2‐distearoyl‐sn‐glycero‐3‐phosphatidylethanolamine‐PEG carrying mannose derivatives to improve nanoparticle retention in lymph nodes for efficient antigen stimulation. The polyethylene glycol moiety itself served to stabilize the nanoparticles and to avoid rapid clearance from the body. Also, the potent PAMP CpG (a TLR9 agonist) was encapsulated in the CaP‐nanoparticle’s core together with the peptide antigen. Immunization with these nanoparticles significantly reduced liver metastasis in CT26 mice tumor models through the generation of a strong CTL immune response.

Recently, the synthesis of biodegradable nanoparticles made of nonmannosylated and mannosylated polylactic‐co‐glycolic acid/polylactic acid has been described [157]. These nanoparticles were designed for the simultaneous *in vivo* delivery of melanoma‐derived antigens and different Toll‐like receptor ligands like CpG (TLR9) and MPLA (TLR4). This strategy allows targeting of DCs *via* a passive phagocytosis‐dependent mechanism and in parallel via an active ligand‐mediated targeting through the mannose receptor. These nanoparticles were able to entrap CpG and MPLA within the same polymeric matrix allowing the simultaneous co‐stimulation of different TLRs. In addition, this study reported the synergism of mannosylated polymers with PD‐1/OX40 immune checkpoint therapy in mouse models, showing long‐term survival and diminished melanoma growth.

#### Development of glycan‐directed CAR‐T cells

**Fig. 3:**
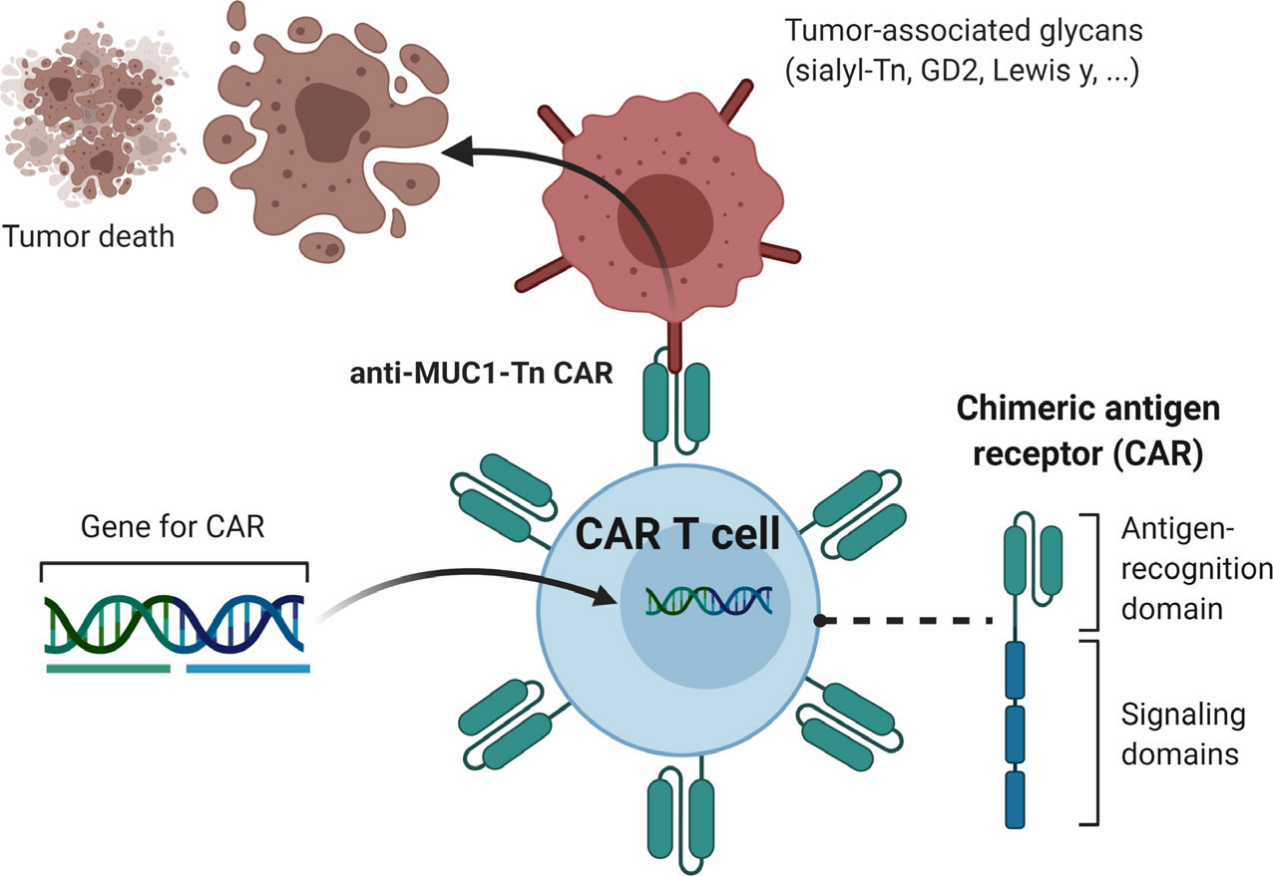
CAR‐T represent another strategy to target distinct glycan epitopes, unique for tumor cells.

Besides the use of glycans as antigen carriers, antiglycan antibodies offer a unique opportunity to develop glycan‐directed CAR‐T cells (Fig. 3). CAR‐T cells are T cells that through genetic engineering express artificial antigen receptors, consisting of transgenic constructs that encode Fab antibody fragments on their cell surface, linked to a transmembrane region and intracellular signaling motifs. The specificity for a given antigen is secured by the Fab regions derived from heavy and light chains of a respective antibody directed to that antigen, and the intracellular signaling domains, usually from CD3zeta, CD28, or 4‐1BB, to facilitate T‐cell activation and cytotoxicity [158]. The recent success and FDA approval for CAR‐T therapy in B‐cell leukemia has inspired researchers to develop novel CAR‐T approaches directed against solid tumors and infectious agents. In this respect, tumor‐associated glycans may offer unique opportunities for CAR‐T design [159, 160], whereby the antigen specificity of a carbohydrate‐specific antibody is coupled to the T‐cell activation machinery and inserted to generate the glycan‐directed CAR‐T. The first‐generation carbohydrate‐specific CAR‐T cells were developed ~ 20 years ago and were directed against the tumor‐associated glycans sialyl‐Tn [161, 162], GD2 [163], and Lewis y [164]. Although some of these glycans might also be expressed in healthy tissues, the first clinical trial using the Lewis y CAR‐T showed that the glycan CAR‐T cells displayed some clinical benefit without high‐grade toxicity [165]. Interim results from a phase 1 clinical trial employing GD2‐CAR‐ natural killer T (NKT) cells demonstrated that the GD2‐CAR‐NKT cells expanded *in vivo*, homed to the neuroblastoma tumors, and in one patient even caused an objective response [166].

Two CAR‐Ts have been developed that recognize the MUC1 protein carrying Tn antigen [167, 168], widely expressed in adenocarcinomas and also on blasts of acute myeloid leukemia. The MUC1‐Tn CAR‐Ts were reactive against multiple tumor types *in vitro* and also demonstrated clear antitumor efficacy in a xenograft model of pancreatic cancer. Recently, a more sophisticated Tn‐specific CAR‐T was constructed, using an antibody (237Ab) fragment specific for a Tn‐containing glycopeptide in podoplanin [169]. This 237Ab was further optimized showing enhanced affinity for both the Tn‐podoplanin, as well as the Tn‐MUC1 [170]. The CAR‐T cells configured with the optimized antibody displayed a broader cross‐reactivity and more efficient reactivity against *O*‐glycosylation‐defective murine and human tumor cell lines *in vitro*. In addition, a novel chondroitin sulfate proteoglycan 4 CAR‐T could control tumor growth in glioblastoma preclinical models. [171]

Applications of glycan‐directed CAR‐T stretch way beyond tumor therapy and may also be useful in the treatment of (chronic) infectious diseases (for an overview, see Ref. [160]). Especially, the HIV virus has served as a target in the CAR‐T field, with researchers designing chimeric receptors that bind the surface antigens exposed on infected cells. Ghanem *et al*. used bispecific CARs based on both the CD4‐gp120 and C‐type lectin‐high‐mannose patch interactions [172]. In their hands, the CD4‐MBL bispecific CAR‐T outperformed the CD4‐DC‐SIGN CAR‐T, displaying an enhanced potency toward different HIV strains. Recently, a CAR‐T using the Dectin‐1 binding domain to engage β‐glucans on *Aspergillus fumigatus* has been developed as well [173]. These Dectin‐1 CAR‐T cells efficiently bound the germinating fungi and their application led to reduced fungal lesions *in vivo*.

Although there is a long way to go before the glycan‐directed CAR‐T cells are approved for mainstream therapy, they show great promise in the fight against cancer and also infectious diseases and are important new directions in the CAR‐T cell field.

In a literal way, glycosylations can be exploited to ‘steer’ the trafficking patterns of CAR‐T cells within the body. On the surface of leukocytes, expression of the tetrasaccharide sLe^x^, the canonical‐binding determinant for selectins, mediates the migration of these cells to E‐selectin^+^ endothelial beds. All CAR T cells commercially produced to date lack surface expression of sLe^X^, but they characteristically display sialylated type 2 lactosamines. Accordingly, α(1,3)fucosyltransferase‐mediated cell surface exofucosylation of the CAR‐T cell sialylated type 2 lactosamine acceptors engenders sLe^x^ expression, and these cells are then capable of migrating with high efficiency to tissue beds that express E‐selectin [174, 175]. Since E‐selectin expression is constitutive within marrow microvessels and is also characteristic of tumor vascular beds, exofucosylated CAR‐T cells can enter marrow with greater efficiency (i.e., for CAR‐T cell therapy directed against leukemias, lymphomas, and multiple myeloma) and would also have heightened infiltration of tumor beds in solid malignancies. By increasing the capacity of administered CAR‐T cells to colonize relevant sites of malignancy, CAR‐T cell surface glycoengineering would decrease the cell dose needed to achieve a desired therapeutic effect and, commensurately, would decrease the costs required for *in vitro* expansion of CAR‐T cells [175].

### Suppression of the immune response

The immune system is often generalized as the body’s defenses against foreign and aberrant tissues, cells, and molecules. It can be evolutionary conserved, as in the innate immune system, building on genetically inherited immunity to antigenic structures carried by pathogens that are not widely represented in self‐tissues. As pathogens rapidly modify their antigens to circumvent innate immune attack, higher organisms have developed an adaptive immune system consisting of lymphocytes with a unique repertoire of antigen receptors produced by somatic mutations and genetic recombinations that complement the diversity of pathogens. However, the other side of the coin is that the immune system may also attack self‐tissues.

Autoimmune diseases are heterogeneous group of ~ 80 complex diseases characterized by the loss of immunological tolerance to self‐antigens. Autoreactive antibodies damage tissues that can target and affect the condition of single or even multiple organs. The ethology of autoimmune diseases is in most common cases not fully understood and is believed to be a mix of predisposing genetic factors, environmental conditions, and bacterial, viral, or fungal infections.

From general point of view, a (very) limited part of the self‐antigen heterogeneity is represented by proteins, whereas the major structural variations are represented by carbohydrates and to some extent by lipids. T cells can, however, interact with posttranslational modifications, such as glycans, on the side chains of the peptide. This is of particular importance as this could represent new and variable epitopes that in self‐tissues could also be targets for the immune system and cause autoimmune diseases.

Microbial infections have long been considered as one of the risk factors of autoimmune disease. This molecular mimicry is defined as antigenic similarity between bacterial and host molecules, including glycans, whereby humoral responses against bacterial oligosaccharides or peptides result in autoantibodies and pathological inflammatory reactions and may even trigger autoimmunity. Moreover, bacterial oligosaccharides mimicking human cell surface glycans may hamper humoral and cell‐dependent immune responses [3, 176]. In addition, selected bacterial species have developed strategies to avoid the host's immune system by covering their cell wall surfaces by oligosaccharides similar to that of the host organism. Molecular mimicry has been described for *Helicobacter pylori* [177], *Streptococcus pyogenes, S. agalactiae, Haemophilus influenzae* [178], *Neisseria meningitidis, N. gonorrhoeae* [179], and *Campylobacter jejuni* strains [180]. *C jejuni* expresses on its capsule lipooligosaccharide mimics of ganglioside residues. Antibodies against *C. jejuni* cross‐react with host neuronal gangliosides causing serious neurological disorders, such as autoimmune‐based Guillain–Barré syndrome (GBS) [180]. *Mycoplasma pneumoniae*, the cause of respiratory tract infections, has also been linked to GBS through molecular mimicry. Anti‐galactocerebrosides antibodies directed against glycolipids from *M. pneumoniae* cross‐react with the main glycolipids in the myelin of both the central and peripheral nervous system. Molecular similarity between the GM1 ganglioside and *M. pneumoniae* glycolipids has also been described [181].

#### The role of glycans in autoimmune diseases: the case of rheumatoid arthritis

An intriguing case of a glycan‐dependent autoimmune response is found in rheumatoid arthritis [182]. As recently reported, one of the few defined glycosylated target epitopes is located on type II collagen. Interestingly, there is a wide repertoire of different TCR in both mice and humans that specifically interact with this glycosylated side chain [183, 184]. T‐cell recognition of this epitope is the critical bottleneck for development of collagen‐induced arthritis in mice [185] and is also of relevance in rheumatoid arthritis [182]. The major T‐cell recognition site is the side chain of a lysine at position 264, which can become hydroxylated, and if this occurs, hydroxylysine can become glycosylated with either a mono‐ or a disaccharide (galactose and glucose). Indeed, immunization of mice (expressing the mouse Aq or the human DR*0401) with collagen type II activates T cells that preferentially recognize the glycosylated lysine side chain.

T‐cell recognition of collagen type II thus represents a unique example of carbohydrate recognition that could be of fundamental importance for both immune selection and for tolerance induction and thus the vaccination of rheumatoid arthritis. Interestingly, collagen type II is one of several tissue‐specific antigens that are expressed in the thymus. It is expressed in medullary thymic epithelial cells [186], which is an antigen‐presenting cell that presents endogenous peptides to the developing T cell [187]. After an antigen‐specific interaction with the medullary thymic epithelial cell, T cells are either ignored, deleted, or differentiate into regulatory T cells. These medullary thymic epithelial cells express a fully native triple helical collagen type II, otherwise expressed only in chondrocytes. However, in contrast to the chondrocyte [188] it lacks glycosylation. Consequently, T cells specific for the glycosylated side chain on the lysine at position 264 escape negative selection. Thus, due to the glycosylation of collagen type II, T cells cannot be completely tolerized in the thymus, hence representing an increased risk of autoimmune disease.

The glycosylation of collagen type II does not only provide a risk but also an opportunity. Induction of tolerance through the exposure to tissue antigens might be easier to manipulate than the centrally regulated (i.e., thymus) induction of tolerance. In fact, it is clear that the glycosylated epitope on collagen type II represents an epitope detected by regulatory T cells and could induce tolerance [189]. It has been shown that intravenous injection of complexes of the MHC class II molecule and the glycosylated CII peptide was able to prevent the development of arthritis and could also suppress already established disease [190, 191]. Treatment with a nonglycosylated collagen type II peptide had no effect. Interestingly, the vaccination effect was dominant as it could be transferred using T cells and is due to a strengthened peripheral tolerance, that is, the critical physiologic mechanism giving protection from an overactivated self‐reactive immune system. In conclusion, the T‐cell recognition of *O*‐linked glycosylation could play a critical role in protecting us from autoimmune disease.

Taken together, T‐cell recognition of collagen type II represents a unique recognition of carbohydrates that could be of fundamental importance for both selection of the immune system and for tolerance induction [186] and thereby vaccination of rheumatoid arthritis [190]. Thus, redirecting the glycosylation of antigens can effectively edit their immunogenicity toward more tolerogenic responses, which might be harnessed in future therapies to cure allergies or autoimmune diseases.

#### Immunomodulation by mono‐ and multivalent carbohydrate conjugates

**Fig. 4:**
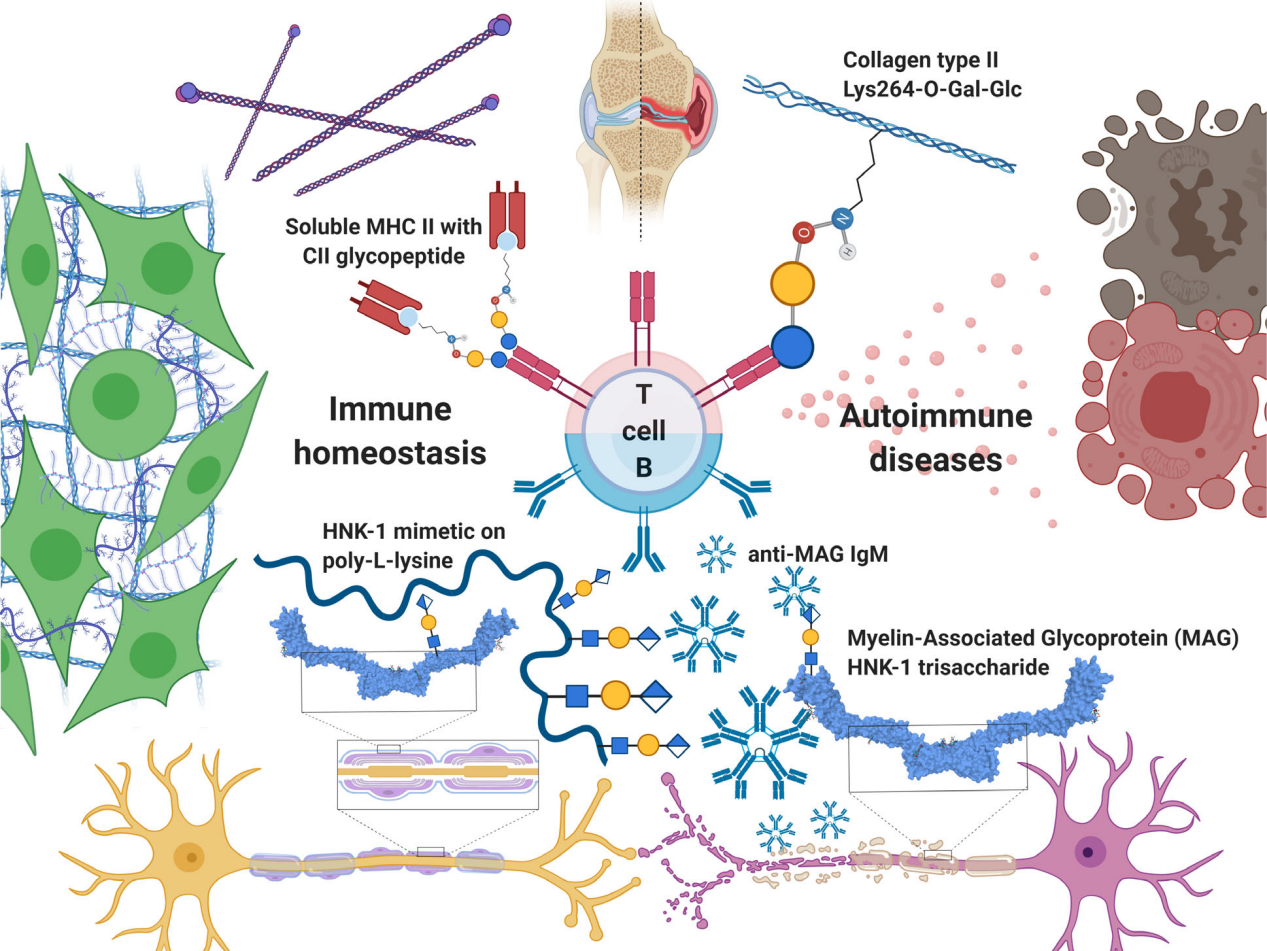
Glyco‐based strategies to modulate immune responses. The fragile balance between immune homeostasis and autoimmune diseases can be guided by glycan epitope‐containing reagents. In case of collagen type II‐induced arthritis, T cells can be educated to tolerate the tissue by using glycopeptide‐loaded soluble MHC II complexes displaying the troublemaking glycan epitope, whereas in the case of anti‐MAG neuropathy, anti‐MAG IgM antibodies are scavenged by HNK‐1 glycopolymer conjugates.

Just very recently, a novel strategy for modulating immune responses by multivalent carbohydrate conjugates was presented by Herrendorff *et al*. [192] This methodology was successfully implemented in an *in vivo* model for anti‐myelin‐associated glycoprotein (MAG) neuropathy with an autoimmune etiology, where high titers of the IgM anti‐MAG antibodies are unequivocally associated with myelinated nerve fiber demyelination (Fig. 4) [193, 194]. IgM anti‐MAGs recognize human natural killer‐1 (HNK‐1) trisaccharide epitope SO_3_‐3‐GlcA(β1–3)Gal(β1–4)GlcNAc, which is highly expressed on MAG (Fig. 4). Upon recognition, IgM anti‐MAGs recruit complement and provoke MAG phagocytosis with concomitant direct inhibition of MAG’s adhesion and signaling functions. The present therapies for anti‐MAG neuropathy, using nonspecific immunosuppressives, act to reduce anti‐MAG antibodies, but due to a lack of selectivity lead to severe side effects [195]. Herrendorff *et al*. constructed a multivalent glycoconjugate with HNK‐1 mimics bound to repetitive ε‐amino groups of a poly‐l‐lysine biodegradable support (Fig. 4). The multivalent glycoconjugate inhibited binding of IgM anti‐MAG antibodies to MAG at low to sub‐nanomolar inhibitory constants, as measured in patients’ sera. Furthermore, *in vivo* data clearly demonstrate the efficient removal of pathogenic anti‐MAGs in a mouse model for anti‐MAG neuropathy.

We also point to another molecule in the paradigm how immunomodulation can be achieved by small carbohydrate‐based molecules. Probably, the most advanced molecule in this field is eritoran (Fig. 4), a mid‐sized glycolipid conjugate currently in phase III clinical trials as a specific immunosuppressant [196, 197]. Bacterial lipopolysaccharide lipid A is a PAMP that binds to pattern recognition receptor TLR4 and triggers the release of inflammatory mediators that contribute to septic shock by inducing severe vasodilation, capillary leakage, and pulmonary hypertension. By mimicking lipid A without exerting TLR4 activation, eritoran selectively antagonizes the TLR4‐mediated excessive reaction during infections. Intriguingly, its effects have not been confirmed in sepsis shock treatment, but it is currently being evaluated for influenza‐associated cytokine storm treatment.

#### The role of glycans in immune suppression: the Siglec case

**Fig. 5:**
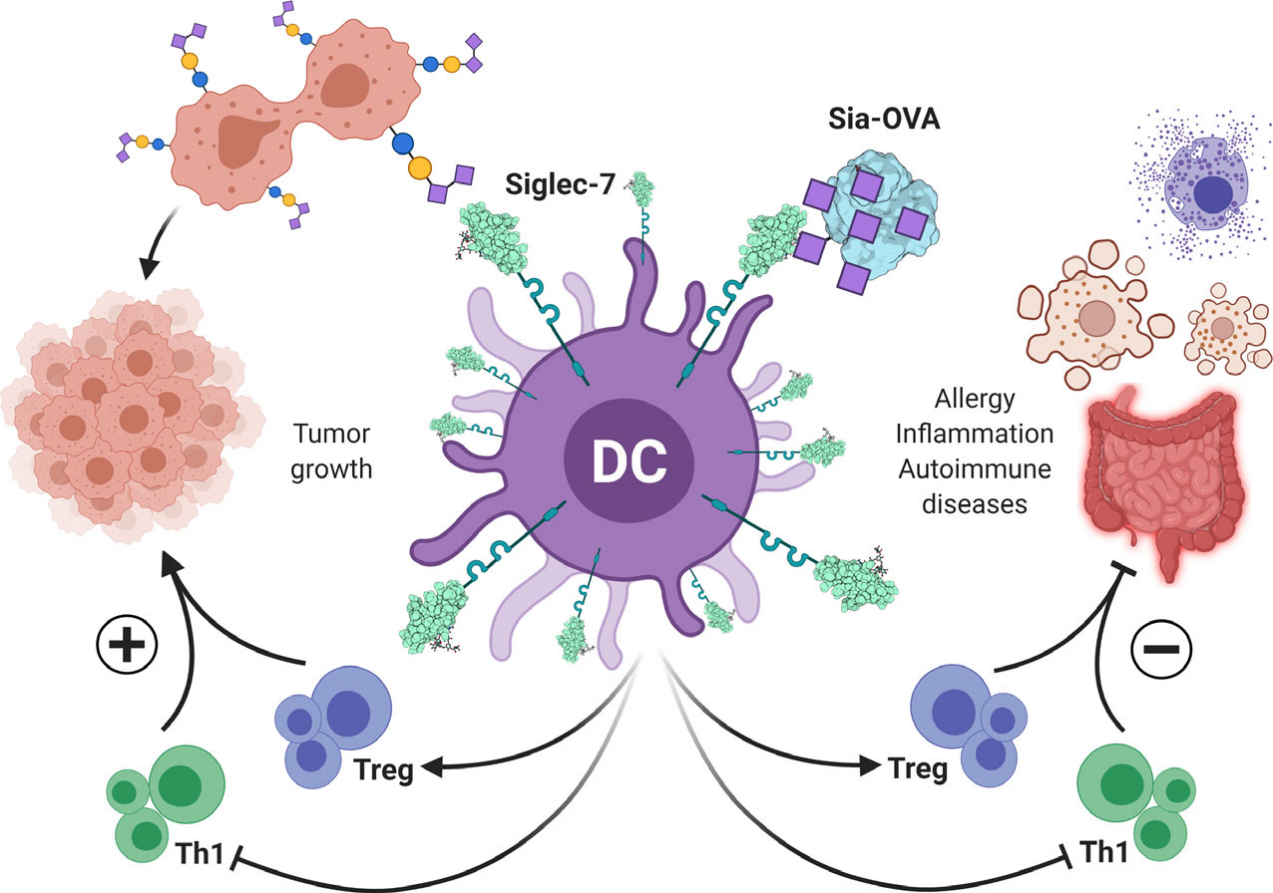
Sialylated tumor‐associated glycans can dampen antitumor immune response through interaction with Siglecs, a family of inhibitory receptors found on DCs. Through the interaction with, for instance, Siglec‐7. DCs instruct the differentiation of Treg, while T helper 1 (Th1) responses are inhibited, together promoting tumor growth (left). On the other hand, Siglec‐mediated inhibition might be harnessed to prevent overactive immune response in allergy, chronic inflammation, and autoimmune disease settings, for example, by using artificially designed sialylated polymers or conjugates to control unwanted T helper responses and to promote Treg differentiation (right).

Of particular interest in the inhibition of unwanted or exacerbated immune reactions are sialic acids, which decorate the majority of mammalian glycans at the cell surface and in the extracellular space and are therefore often considered as SAMPs [26, 198]. Sialylated structures are known to interact with sialic acid‐binding immunoglobulin‐like receptors or Siglecs (Fig. 5). This family of lectin receptors is highly expressed within the immune system, especially on myeloid cells (DC, macrophages) and on NK cells [199] and many of them contain immunoreceptor tyrosine‐based inhibitory motifs (ITIMs) within their cytoplasmic domains, allowing them to recruit phosphatases, thereby increasing the threshold for activation.

Particularly, bacteria commonly use host sialic acid residues to mimic host glycosylation patterns. Due to the widespread distribution on the host mucosal surface as well as the cellular glycocalyx, acquisition of sialic acid provides a mechanism of avoiding adaptive immune recognition, thereby facilitating pathogenic bacterial survival [199]. For instance, *S. agalactiae* can produce its own sialic acid‐capped structures that are able to engage inhibitory Siglecs, preventing host responses and suppressing pro‐inflammatory cytokine release, oxidative burst, neutrophil extracellular trap production, and phagocytosis. Another example of molecular mimicry is sialylated glycans present on the cell wall of group B *Streptococci* that are capable of inhibiting the complement system by reducing the deposition of C3b fragments on their surface and consequently preventing the membrane attack complex, cumulatively enhancing Group B *Streptococcus* survival [200, 201, 202].

Also, tumor cells are often hypersialylated, which through the interaction with Siglec receptors leads to immune evasion in, for example, non‐small‐cell lung cancer, colorectal, and ovarian cancer patients [203] as well as in multiple *in vivo* murine colorectal, melanoma, and lung cancer models [204, 205]. Indeed, many research groups focus on abolishing this detrimental sialic acid‐Siglec axis in cancer through sialic acid or Siglec blockade to relieve the immune evasion. These strategies involve the design of specific inhibitors or sialic acid mimetics that can block the relevant sialyltransferases or *de novo* sialic acid synthesis in the tumor [204, 206]. Alternatively, the sialic acids on the tumor may be enzymatically removed by targeting tumor cells using tumor‐specific antibodies (anti‐HER2) coupled to a sialidase, which subsequently cleaves the sialic acids, thereby releasing the brakes on the immune system [207]. Next to suppressing sialic acid expression in the tumor, other approaches focusing on the immune inhibitory Siglecs and specific small molecule inhibitors of Siglec‐7 on NK cells have already been developed [208].

Nevertheless, the immune suppression mediated by the sialic acid‐Siglec engagement might also be harnessed to actually suppress immune responses. Bertozzi and co‐workers developed sialoside glycopolymers that contain the Siglec‐7 ligand GD3. These polymers can effectively be incorporated in cells, thereby inhibiting NK cell‐mediated cytotoxicity through the engagement of Siglec receptors on the NK cell surface [209]. Proof of principle was also provided by the work of Perdicchio *et al*. [210, 211] Through chemical coupling the authors decorated the model antigen OVA with sialic acids, which were able to reprogram DC responses and inhibit pro‐inflammatory T helper 1 responses and facilitating *de novo* differentiation of regulatory T cells both *in vivo* and *in vitro* in a Siglec‐E‐dependent manner. Poly(lactic‐co‐glycolic acid) nanoparticles decorated with α2‐8 sialic acids have strong anti‐inflammatory properties and could inhibit pathology in several LPS‐induced sepsis models through engagement of Siglec‐E and by augmenting IL‐10 secretion in mouse macrophages [212].

The restricted expression of CD22 (Siglec‐2) on B cells has inspired researchers to target this receptor for instructing B‐cell tolerance or to dampen harmful B‐cell responses allergy or autoimmunity. By incorporating high‐affinity CD22 ligands (^BPA^NeuGcα2‐6Galβ1‐4GlcNAc) in antigenic liposomes, antigen‐specific B cells go into apoptosis and are thus deleted from the repertoire [213]. Indeed, these so‐called STALs (Siglec‐engaging tolerance‐inducing antigenic liposomes) could prevent sensitization to the peanut allergen Ara h 2 [214]. Similarly, rapamycin‐incorporated STALs, using OVA as a model antigen, were only effective prior to sensitization and showed little efficacy in previously sensitized mice [215]. Proof of concept for the treatment of autoimmune disease was provided by the study of Bednar *et al*. [216] STALs containing the human CD22 ligand 6′MBP‐5F‐Neu5Ac and synthetic cyclic citrullinated peptides could prevent *in vitro* production of autoantibodies by B cells isolated from rheumatoid arthritis patients. Also *in vivo*, STALs containing the citrullinated peptides tolerized mice inhibiting subsequent autoantibody production to citrullinated peptide challenge. Overall, sialic acid‐containing liposomes are a promising new tool to abolish unwanted B‐cell immunity, yet further improvements are required to reach their full potential in already sensitized individuals. A newly developed humanized CD22 transgenic mouse model, constructed by knock‐in of the extracellular human CD22 coupled to the intracellular domains of murine CD22, will be of great help in the preclinical assessment of novel STAL formulations [217].

Besides CD22, other Siglecs, such as Siglec‐8 and CD33 (Siglec‐3), form attractive targets for glycan‐based immunotherapy of allergic responses. Siglec‐8 has a very restricted expression pattern and is only present on eosinophils, mast cells and to a lesser extent on basophils. The anti‐Siglec‐8 antibody AK002 (lirentelimab) has shown promising results in a phase 2 clinical trial inhibiting gastrointestinal symptoms in patients with eosinophilic gastritis or duodenitis [218]. High‐affinity sialic acid‐based ligands have now been identified for Siglec‐8 (6′‐O‐sulfo NSANeu5Ac) [219], paving the way for glycan‐based targeting of Siglec‐8. CD33 is highly expressed on mast cells and thus could be employed to counteract mast cell degranulation. Indeed, liposomes loaded with trinitrophenol as the model antigen and a high‐affinity sialic acid analogue ligand for CD33 bound human mast cells in a CD33‐dependent manner and could inhibit IgE/FcεRI signaling, as well as IgE‐mediated anaphylaxis in human CD33 transgenic mice *in vivo* [220].

Together, these results indicate that it might be achievable to develop nanovaccines that instead of activating immunity actually dampen unwanted immune responses in sepsis, allergy, and autoimmunity, thus extending the repertoire of vaccine modalities and functional approaches.

## Final considerations and future perspective

In conclusion, the combination of nanotechnology and glycans signifies an added value in the constant effort to achieve effective carbohydrate‐based cancer treatments. The importance of controlling the antigen display and the insertion of different epitopes and adjuvants on the same formulation seem to point out that controlled nanoengineering could help at obtaining universal platforms for fully or semi‐synthetic glycovaccines. A rational design of C‐type lectin‐targeting glycans and their configuration is of utmost importance to obtain the optimal vaccine formulation. This is a crucial point that must be taken into account in antigen‐presenting cell‐targeting glycan‐based vaccines. However, the importance of checking (nano)toxicity should be a priority together with a quality by design‐like approach in order to foresee from the beginning whether the concrete nanosystems are suitable for scale‐up and GMP production.

Vaccine development using glyco‐decorated gold nanoparticles, poly‐ or oligosaccharide conjugates, glycosylated nanovaccines, and specific glycopeptides and glycoproteins, all aim to achieve a better antigen presentation or better antigen delivery. However, strategies aimed at modulating immune response go well beyond this approach and may even involve a dampening of immune reactions in autoimmunity or allergic diseases. We have highlighted some case‐stories, with high potential, including the use of glycopeptides to induce tolerance to collagen type II in RA, as well as the use of sialic acids or HNK‐1 mimics to dampen unwanted immune responses.

The advancements of the last decade in glycan analytics and glyo‐gene engineering will open up new avenues to explore the contribution of glycans in autoimmune and antitumor immune responses. Moreover, it will expand our knowledge on the expression of autoimmune and tumor‐specific glycan structures, their recognition by lectin receptors, and the subsequent immune modulation through lectin signaling. Finally, the development of specific glycan moieties or glyco‐mimetics will enable the specific targeting of individual lectin receptors, taking advantage of their intrinsic ability to facilitate antigen uptake and processing and to steer immune responses. This efforts require a continuous communication between immunologists and glyco‐chemists, which we envision will propagate the design and development of the novel glycan‐based immunotherapies of the future.

## Conflict of interest

The authors declare no conflict of interest.

## Author contributions

MA, FB, ABW, FC, CC, FC, KD, XF, RH, DJ, WK, LL, MMC, MM, MO, LP, JJRM, CR, RS, AS, US, OV, FY, BR, SvV conceptualized and wrote the manuscript. OV designed figures; and BR and SvV finalized the manuscript.
